# Kaposi sarcoma in anti-neutrophil cytoplasmic antibody-associated vasculitis: a case-based review

**DOI:** 10.1007/s00296-021-04810-w

**Published:** 2021-02-23

**Authors:** Benedict K. Tiong, Arun S. Singh, G. Peter Sarantopoulos, Tanaz A. Kermani

**Affiliations:** 1 Division of Rheumatology, David Geffen School of Medicine, University of California, Los Angeles, 2020 Santa Monica Boulevard, Suite 540, Santa Monica, CA 90404 USA; 2 Division of Hematology and Oncology, David Geffen School of Medicine, University of California, Los Angeles, Los Angeles, CA USA; 3 Department of Pathology and Laboratory Medicine, UCLA Medical Center, David Geffen School of Medicine, University of California, Los Angeles, Los Angeles, CA USA

**Keywords:** Anti-neutrophil cytoplasmic antibody-associated vasculitis, Microscopic polyangiitis, Granulomatosis with polyangiitis, Kaposi sarcoma, Prednisone, Vasculitis

## Abstract

Anti-neutrophil cytoplasmic antibody-associated vasculitis (AAV) are systemic necrotizing vasculitides associated with significant morbidity and mortality. Given the immunosuppression used to manage these conditions, it is important for clinicians to recognize complications, especially infectious ones, which may arise during treatment. Kaposi sarcoma (KS) is a lymphoangioproliferative neoplasm caused by human herpes virus 8 (HHV-8). Its cutaneous manifestations can mimic vasculitis. We describe a 77-year-old man with microscopic polyangiitis with pulmonary-renal syndrome treated with prednisone and intravenous cyclophosphamide who developed KS (HHV-8 positive) after 2 months of treatment. Cyclophosphamide was discontinued and prednisone gradually lowered with improvement and clinical stabilization of KS lesions. This comprehensive review includes all published cases of KS in patients with AAV, with a goal to summarize potential risk factors including the clinical characteristics of vasculitis, treatment and outcomes of patients with this rare complication of immunosuppressive therapy. We also expanded our literature review to KS in other forms of systemic vasculitis. Our case-based review emphasizes the importance of considering infectious complications of immunosuppressive therapy, especially glucocorticoids, and highlights the rare association of KS in systemic vasculitis.

## Introduction

Anti-neutrophil cytoplasmic antibody (ANCA)-associated vasculitis (AAV) includes microscopic polyangiitis (MPA), granulomatosis with polyangiitis (GPA) (Wegener’s), and eosinophilic granulomatosis with polyangiitis (EGPA) (Churg-Strauss syndrome), all pauci-immune vasculitides which share clinical features and are characterized by the presence of ANCA [1]. Serious organ-threatening disease involvement with rapidly progressive glomerulonephritis, diffuse alveolar hemorrhage but also gastrointestinal, cardiac, and neurologic disease can occur [2]. The standard treatment for severe organ-threatening disease is glucocorticoids, followed by induction therapy with cyclophosphamide, or rituximab [3]. A serious consequence of immunosuppression is opportunistic infections. Kaposi sarcoma (KS) is a lymphoangioproliferative neoplasm that has been associated with human herpesvirus 8 (HHV8) [4–6]. Over the years, there have been four recognized types of KS: classic, endemic, iatrogenic (immunosuppression or transplant associated), and epidemic [4–6]. We report a case of hydralazine-induced AAV with pulmonary-renal syndrome complicated by iatrogenic KS during treatment. We performed a comprehensive search through MEDLINE using the following keywords: Kaposi sarcoma, vasculitis, ANCA vasculitis, granulomatosis with polyangiitis, microscopic polyangiitis, Takayasu arteritis, polymyalgia rheumatica, giant cell arteritis, polyarteritis nodosa, and Behcet's disease. We included articles published in English so that details could be extracted. This resulted in exclusion of 5 non-English articles (1 case in AAV and 4 cases in giant cell arteritis). The references of the individual articles were also examined to find other key references. The aim of the review was to identify common factors which may better aid in identifying patients at risk of this rare complication. Based on our review, glucocorticoids appear to be an important risk factor for KS in patients with vasculitis.

## Case presentation

A 77-year-old man of Italian American descent was referred to us for evaluation of AAV. He presented to an outside facility with a 30-lb weight loss, cough, scant hemoptysis, and worsening dyspnea on exertion. Laboratory evaluation showed acute kidney injury with a creatinine of 2.58 mg/dL (baseline 1.0 mg/dL) and BUN of 30 mg/dL, acute anemia with hemoglobin 6.1 g/dL. During hospitalization, he developed rapidly progressive renal failure requiring initiation of hemodialysis, in addition to gross hemoptysis. Serologies included a positive anti-nuclear antibody (ANA 1:320), positive double-stranded DNA (dsDNA 1:40), p-ANCA of 1:1280, MPO 52 IU (> 1 IU positive), and negative anti-glomerular basement membrane antibody (Table 1). Other serologies including SSA, SSB, Smith, RNP, centromere, Scl-70, DRVVT, cardiolipin, beta-2-glycoprotein, ribosomal P, anti-chromatin antibodies were negative. Histone antibodies were not tested. Bronchoscopy confirmed diffuse alveolar hemorrhage. A kidney biopsy was done but was non-diagnostic, showing acute tubular necrosis and mild mesangial matrix expansion with 4 out of 13 glomeruli which were globally sclerotic with mild parenchymal scarring. The patient was treated for a diagnosis of MPA with pulse does steroids, seven sessions of plasmapheresis followed by intravenous cyclophosphamide 400 mg/m^2^ during hospitalization. He was able to successfully come off hemodialysis after 1.5 weeks with a new baseline creatinine of 2 mg/dL. Four weeks later, he was treated with cycle 2 of intravenous cyclophosphamide 400 mg/m^2^.

**Table 1:** Laboratory findings at initial diagnosis of microscopic polyangiitis (MPA), and, later when diagnosis of Kaposi Sarcoma (KS) was made

Laboratory (reference range)	Value	
	At initial diagnosis MPA	At diagnosis KS
WBC (4.16 – 9.95 × 10E3/uL)	11.5	19.81
Absolute Neutrophil Count (1.80 – 6.90 × 10E3/uL)	9.8	18.22
Absolute Lymphocyte Count (1.30 – 3.40 × 10E3/uL)	0.58	0.68
Hemoglobin (13.5–17.1 g/dL)	6.1	11.6
Platelet Count (143 – 398 × 10E3/uL)	176	259
Sedimentation Rate By Modified Westergren (< OR = 20 mm/h)	> 120	97
C-Reactive Protein (< 0.8 mg/dL)	28.9	1.19
Urea Nitrogen (7–22 mg/dL)	48	86
Creatinine (0.60–1.30 mg/dL)	2.58	2.95
Calcium (8.6–10.4 mg/dL)	9.0	8.2
Phosphorus (2.3–4.4 mg/dL)	3.3	5.5
Total Protein (6.1–8.2 g/dL)	5.8	5.9
Albumin (3.9–5.0 g/dL)	2.2	3.1
Alkaline Phosphatase (37–113 U/L)	43	217
Aspartate Aminotransferase (13–47 U/L)	28	88
Alanine Aminotransferase (8–64 U/L)	18	58
Procalcitonin (< 0.10 ug/L)	1.53	8.30
Immunoglobulin G serum (nl 726–1521 ml/dL)	Not tested	533
Immunoglobulin A serum (nl 87–426 ml/dL)	Not tested	201
Immunoglobulin M serum (nl 44-277 ml/dL)	Not tested	92
HIV	Negative	Negative
dsDNA Ab	Positive, 1:40	Negative
C-ANCA (< 1:20 titer)	Negative	Negative
P-ANCA (< 1:20 titer)	1:1280	Negative
Proteinase-3 Ab	Negative	Negative
Myeloperoxidase Ab	52 (> 1 positive)	< 20.0 (> 20 positive)
C3 (76–165 mg/dL)	126	132
C4 (14–46 mg/dL)	34	35
Urinalysis		
Protein/Creatinine Ratio,Ur (0.0–0.4)	0.82	0.3
RBC per HPF (0–2 cells/HPF)	> 20	0
WBC per HPF (0–4 cells/HPF)	0–2	0
Hyaline Casts (0–2/LPF /LPF)	0	11–20

**Fig. 1:**
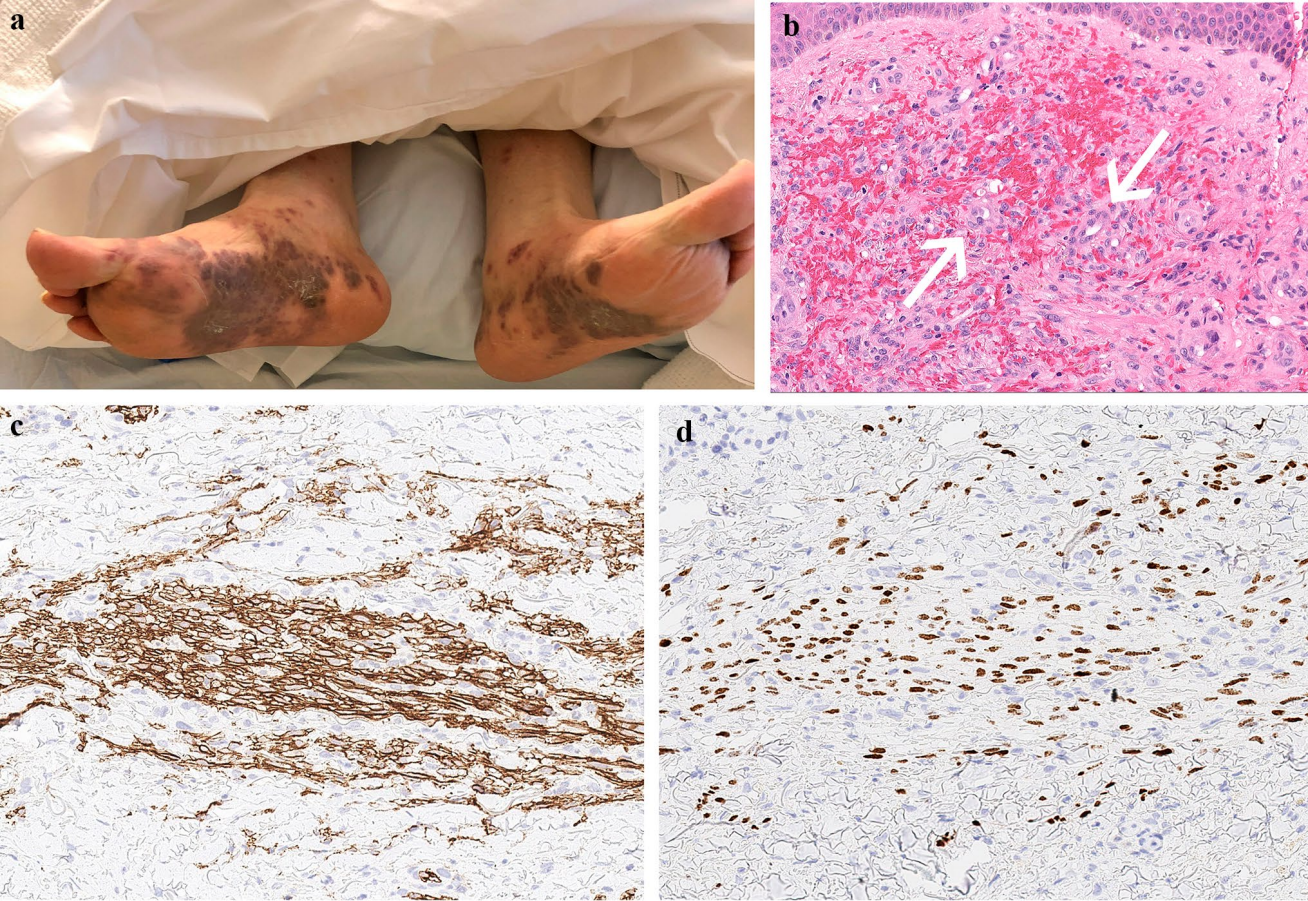
**a** Multiple violaceous, coalescent, nodular lesions on the foot and ankle. **b** Histologic sections of skin from biopsy of a thigh lesion show dermis filled with irregular, somewhat jagged blood-filled vascular spaces adhering to collagen bundles and surrounding native blood vessels (so-called ‘promontory sign’, see arrows). *Hematoxylin and eosin, 200x*. **c** Performed CD34 immunohistochemistry strongly highlights irregular, infiltrative vascular spaces. *CD34 immunohistochemistry, 200x*. **d** Performed HHV8 immunohistochemistry highlights tumor endothelial cell nuclei and confirms the diagnosis of Kaposi’s sarcoma. *HHV8 immunohistochemistry, 200x*

Approximately 2 months after starting treatment, he developed a new lower extremity rash (Fig. 1a). Prednisone was increased from 40 to 45 mg by his local rheumatologist with some improvement. However, given persistent symptoms, the patient sought a second opinion at our tertiary care medical center. At the time of evaluation, he was on prednisone 45 mg daily and last cyclophosphamide (dose 2, 400 mg/m^2^) infusion had been administered 2 weeks prior. Apart from the rash, he denied any symptoms. Laboratory parameters, including renal function, were stable. Medication review revealed that he had been on treatment with hydralazine for hypertension for more than 1 year prior, and, given association of hydralazine with AAV, hydralazine was discontinued. Prednisone was lowered to 35 mg. Rituximab was discussed given severe manifestations of vasculitis but given positive hepatitis B core antibody, recommendation was made for evaluation with infectious diseases first. Unfortunately, 1 month later, he was hospitalized for mental status changes from urosepsis with *Escherichia coli* bacteremia. He was on prednisone 35 mg daily at the time. Treatment was complicated with *Clostridium difficile* colitis. During that hospitalization, further testing was pursued (Table 1). In addition, given lack of improvement in the lower extremity rash, a skin biopsy was obtained from his left thigh and his left foot. This showed an atypical HHV8-positive vascular proliferation without vasculitis consistent with KS (Fig. 1b–d). Testing for human immunodeficiency virus (HIV) was negative. While the initial plan was to start treatment with rituximab based on the severity of the manifestations of vasculitis, given the numerous infectious complications and hospitalizations, along with the absence of any evidence of active vasculitis, the recommendation was to hold off on immunosuppressive therapy. Furthermore, given that there was suspicion of this being hydralazine-induced, it was felt discontinuation of the trigger may also help. After discussion with the different specialists, and the patient, the decision was made to gradually taper prednisone and monitor closely without additional immunosuppressive therapy. He was also referred to oncology for co-management. He did not have any clinical evidence of gastrointestinal mucosal involvement of his KS and was started on treatment with topical imiquimod cream 5%. Chemotherapy was not considered since the etiology of his KS was felt to be due to immunosuppression as well as his recent history of multiple infections, renal insufficiency, and, immunosuppression was being lowered. He remains on prednisone 10 mg daily with adequate control of vasculitis and improvement in skin lesions. He continues to follow with rheumatology and oncology. Since discontinuation of cyclophosphamide and lowering prednisone, hypogammaglobulinemia has resolved with immunoglobulin G of 825 mg/dL (range 600–1540 mg/dl). He has had no further infectious complications and his KS remains clinically indolent.

## Discussion

We present a rare case of MPA, possibly hydralazine-induced, with pulmonary-renal syndrome complicated by the development of KS during treatment with systemic glucocorticoids and cyclophosphamide.

One of the unusual aspects of this case was the possibility of the manifestations of AAV being hydralazine-induced. Severe manifestations including pulmonary-renal syndrome have been described in hydralazine-induced vasculitis [7–12]. A clue to this entity is the serologic profile which often includes other antibodies often seen in systemic lupus erythematosus (which hydralazine can also induce) in addition to ANCA (usually p-ANCA, MPO) [12]. Other positive serologies that have been reported with hydralazine-induced vasculitis are ANA, anti-histone antibodies, positive dsDNA, hypocomplementemia and anti-phospholipid antibodies [9, 12]. Our patient also had positive ANA and low titer dsDNA at initial diagnosis. While other connective tissue disease serologies, complements and testing for anti-phospholipid antibodies was performed, anti-histone antibody was not tested. The possibility of hydralazine-induced vasculitis was missed and considered few months later when he was evaluated at our facility for a second opinion. In most cases of serious organ manifestations, even though hydralazine was thought to be the trigger, in addition to withdrawal of the offending medication, immunosuppressive therapy including glucocorticoids, cyclophosphamide or rituximab were used [7]. In our case, the management was complicated by numerous infectious complications including KS.

The risk factors for KS include infection with HHV8, HIV, and immunosuppression [4–6, 13–15]. Our patient best fits as an example of iatrogenic KS, which has been widely reported in immunosuppressed patients, including those with organ transplantation [5, 16]. Despite the wide-spread use of immunosuppressive medications in systemic rheumatic diseases including vasculitis, the association of KS is rare, indicating there are other risk factors at play [17, 18].

A review of the literature evaluating KS in AAV, identified 10 additional cases whose findings are summarized in Table 2 [19–28]. The majority of the reports (70%) are in patients with GPA, with two reports in MPA and one in EGPA (Table 2). Age range was from 46 to 78 years with 60% of the cases being in men (Table 2). Time to onset of KS lesions ranged from 6 weeks to 11 months (Table 2). One reported case in the literature described coincidental occurrence of KS and GPA with worsening KS during treatment [26]. In another report, KS lesion on an ear was noted 19 years after diagnosis but, unusually, this patient had been given several different inflammatory diagnoses over the years including polyarteritis nodosa, neurosarcoidosis and finally, GPA [19]. All patients were still on systemic glucocorticoids and intravenous methylprednisolone were administered in 80% reported cases (Table 2). Adjunctive immunosuppressive therapy in patients who developed KS included cyclophosphamide (9 cases), azathioprine (3 cases) and mycophenolate mofetil (1 case), likely reflecting commonly used treatments in AAV given most cases had pulmonary and renal manifestations (Table 2). In all except one case, cutaneous involvement from KS was present, most frequently on the trunk and extremities. One case reported isolated KS of the gastrointestinal tract in a patient with GPA [28]. HHV-8 was positive in 6 of 7 cases where the information was provided (Table 2). The majority of cases improved with reduction or withdrawal of immunosuppressive therapy, especially glucocorticoids (Table 2). In some cases, chemotherapy and radiation therapy were also used to treat KS (Table 2). The status of the vasculitis after lowering immunosuppressive therapy was variable with some cases of relapses (Table 2). The patient in this case also presented many management challenges. Though hydralazine was thought to be the likely trigger of vasculitis, given the severe manifestations with pulmonary-renal syndrome, treatment with rituximab was considered. However, the patient had positive hepatitis B core antibody and recommendation was to start prophylaxis prior to treatment. Meanwhile, he developed numerous infectious complications including urosepsis, *Clostridium difficile* requiring hospitalizations which delayed initiation of rituximab. Finally, he also developed infectious complication of KS from HHV-8. Given that there was no evidence of active vasculitis, that hydralazine which may have been a trigger was discontinued, and risks of further immunosuppression, decision was made to use glucocorticoid monotherapy, lower prednisone gradually monitor closely. He clinically improved with reduction in glucocorticoid doses, discontinuation of cyclophosphamide, and topical imiquimod. To date, there has been no recurrence of vasculitis which may be in part from the discontinuation of hydralazine.

**Table 2:** Summary of literature review of ANCA-associated vasculitis with Kaposi sarcoma

Author	Age, years/sex	Diagnosis	AAV Organ involvement	ANCA type	Immunosuppression	Time to onset of KS	Areas affected by KS	HIV and HHV-8 status	Treatment	Outcome
Our case	77/M	Drug-associated MPA	Pulmonary-renal syndrome	p-ANCA, MPO	Glucocorticoids (IV followed by oral prednisone), IV cyclophosphamide	6 Weeks	Skin lesions on upper and lower extremities	HIV negative, HHV-8 positive	Withdrawal of cyclophosphamide, lower prednisone, imiquimod topical	Regression KS, vasculitis in remission
Fatma et al.[20]	72/F	MPA	Pulmonary-renal syndrome	Positive p-ANCA, MPO	Glucocorticoids (IV followed by oral prednisone), IV cyclophosphamide	5 Months	Skin lesions on trunk, lower extremities, face, neck	HIV negative, HHV-8 positive	Withdrawal of immunosuppression	Regression KS, relapse vasculitis with alveolar hemorrhage
Biricik et al.[27]	71/M	MPA	Pulmonary-renal syndrome	p-ANCA positive; MPO/PR3 not tested	Glucocorticoids (IV followed by oral prednisone), IV cyclophosphamide	3 Months	Skin lesions on lower extremities	HIV status not provided, HHV-8 positive	Decrease glucocorticoid dose, cyclophosphamide discontinued, radiation therapy	Regression KS, vasculitis status not provided
Erban and Sokas[21]	78/M	GPA	Pulmonary-renal syndrome, chronic sinusitis, arthralgia	Not tested	Glucocorticoids (oral methylprednisolone), oral cyclophosphamide	10 Weeks	Skin lesions on trunk, upper and lower extremities	HIV negative, HHV-8 status not provided	Glucocorticoid discontinued, cyclophosphamide continued, proton beam radiation to the feet	Regression KS, death from cardiogenic shock during cardiac bypass procedure
Deschenes et al.[22]	54/M	GPA	Sinusitis, cavitary pulmonary lesions	c-ANCA, PR3	Glucocorticoids (IV then oral prednisone), oral cyclophosphamide	8 Weeks	Skin lesions on trunk, upper and lower extremities	HIV negative, HHV-8 status not provided	Glucocorticoids tapered off, cyclophosphamide reduced then discontinued after 20 months	Regression KS, vasculitis in remission
Hoff and Rødevand[19]	46/M	GPA	Cranial neuropathies, sinusitis, arthritis, lung nodules	Negative c-ANCA, p-ANCA MPO/PR3 not tested	Glucocorticoids, IV cyclophosphamide (stopped due adverse effects), methotrexate	~ 19 Years	Skin lesion on ear	HIV and HHV-8 status not provided	None	Died of bladder cancer, vasculitis improved
Bouattar et al.[23]	50/F	GPA	Glomerulonephritis, L nasal ulceration	c-ANCA positive; MPO and PR3 not tested	Glucocorticoids (IV followed by oral prednisone), IV cyclophosphamide	18 Weeks	Skin lesions on trunk, upper and lower extremities	HIV negative, HHV-8 positive	Discontinuation of cyclophosphamide, decrease glucocorticoid dose	Regression KS followed by recurrence, worsening renal function requiring dialysis, death from DIC
Saxena et al.[25]	66/F	GPA	Not provided	Not provided	Glucocorticoids (IV followed by oral prednisone), IV cyclophosphamide	5 Months	Skin lesions on trunk and upper and lower extremities	HIV status not provided, HHV-8 positive	Cyclophosphamide continued for another month then switched to azathioprine, prednisone gradually tapered, azathioprine stopped for worsening KS, IV doxorubicin	Regression KS, vasculitis in remission
Kılıç et al. [26]	70/F	GPA	Nasal septal perforation, glomerulonephritis, pulmonary nodules	c-ANCA positive; MPO/PR3 not tested	Glucocorticoids (IV followed by oral prednisone), IV cyclophosphamide	0 (Present at diagnosis but worse at 12 weeks)	Skin lesions on left lower extremity	HIV negative, HHV-8 negative	Glucocorticoids decreased, cyclophosphamide discontinued, radiation therapy, systemic chemotherapy (treatment not specified)	Not provided
Endo and Nagata[28]	73/M	GPA	Not provided	Not provided	Glucocorticoids (IV followed by oral prednisolone), IV cyclophosphamide 4 cycles) then azathioprine	11 Months	Gastrointestinal ulcerations (upper and lower tract)	HIV negative, HHV-8 positive	Corticosteroids tapered from 11 mg per day to 6 mg per day, azathioprine continued	Ulcerations and lesions improved, vasculitis in remission
Berti et al. [24]	67/M	EGPA	Glomerulonephritis, sinusitis, asthma, nasal polyposis	Not provided	Glucocorticoids (oral), mycophenolate mofetil	Not provided	Cutaneous	HIV negative, HHV-8 positive	Mycophenolate mofetil was discontinued, prednisone continued (5 mg per day)	Regression KS, vasculitis in remission

*AAV* anti-neutrophil cytoplasmic antibody-associated vasculitis, *ANCA* anti-neutrophil cytoplasmic antibody, *EGPA* eosinophilic granulomatosis with polyangiitis, *F* Female, *GPA* granulomatosis with polyangiitis, *HHV-8* human herpesvirus 8, *HIV* human immunodeficiency virus, *IV* intravenous, *KS* Kaposi sarcoma, *M* male, *MPA* microscopic polyangiitis, *MPO* myeloperoxidase, *PR3* Proteinase 3

Given the rarity of KS in AAV, we also extended our literature review to other forms of systemic vasculitis (Table 3). We found reports in giant cell arteritis (4 cases), Behcet’s disease (2 cases), polymyalgia rheumatica (3 cases), IgA vasculitis (previously Henoch-Schonlein purpura, 1 case), and cutaneous vasculitis (1 case) [17, 29–37]. In all cases, patients were on glucocorticoid therapy (Table 3). As in the case of patients with AAV, cutaneous involvement from KS was present, most frequently on the trunk and extremities. There was a case of systemic involvement with KS of the gastrointestinal tract in a patient with Behcet’s disease [34]. The majority of cases improved with reduction or withdrawal of immunosuppressive therapy, especially glucocorticoids, with some relapses of the underlying vasculitis in some cases (Table 3).

**Table 3:** Literature review of Kaposi Sarcoma (KS) in other forms of systemic vasculitis

Author	Age, years/Sex	Diagnosis	Immunosuppression	Time to onset of KS	Areas affected by KS	HIV and HHV8 status	Treatment	Outcome
Klepp et al. [17]	79/F	Polymyalgia rheumatica	Glucocorticoids (oral prednisone)	7 Months	Skin lesions lower extremities and eyelid	HIV status not provided, HHV-8 status not provided	Radiotherapy	Regression of KS, patient died suddenly of unknown cause
Vincent et al. [33]	84/F	Polymyalgia rheumatica	Glucocorticoids (oral prednisone)	4 Months	Skin lesions on lower extremities	HIV status not provided, HHV-8 positive	Not provided	Not provided
Brambilla et al. [37]	72/F	Polymyalgia rheumatica	Glucocorticoids (oral prednisone)	4 Years (was on 4 mg daily for 4 years)	Skin lesions on trunk, upper and lower extremities, leg lymphedema	HIV negative, HHV-8 positive	Gradually discontinue prednisone, taxol	Partial regression of KS, developed Merkel cell carcinoma requiring additional treatment
Leung et al. [29]	70/F	Giant cell arteritis	Glucocorticoids (oral prednisone)	5 Months	Skin lesions upper and lower extremities, neck, lips, back	HIV status not provided, HHV-8 status not provided	Decrease in prednisone doses	Regression of KS, no flares of giant cell arteritis
Di Giacomo et al. [30]	69/M	Giant cell arteritis	Glucocorticoids (oral prednisone)	3 Months	Skin lesions lower extremities	HIV status not provided, HHV-8 status not provided	Decrease in prednisone, change to methyl-fluoro-prednisolone	Status of KS not available, flare of giant cell arteritis
Soria et al. [32]	45/F	Giant cell arteritis	Glucocorticoids (oral prednisone)	3 Years	Skin lesions upper and lower extremities, face, trunk	HIV status not provided, HHV-8 status not provided	Decrease in prednisone, vincristine, radiation therapy	Regression of KS, status of giant cell arteritis not provided
Kuttikat et al. [35]	79/F	Giant cell arteritis	Glucocorticoids (oral prednisolone)	6 Weeks	Skin lesions on trunk, lower extremities	HIV negative, HHV-8 positive	Taper of prednisone with discontinuation	Resolution of KS, no flares of giant cell arteritis
Kotter et al. [34]	29/M	Behcet’s disease	Glucocorticoids (oral prednisolone), cyclosporine A, azathioprine	3 Years	Skin, gastric mucosa, hard palate, pulmonary	HIV negative, HHV-8 positive	Discontinuation of azathioprine and cyclosporine A, taper prednisolone	Ocular disease flared requiring treatment with interferon alpha, both diseases in remission
Mezalek et al. [36]	44/M	Behcet’s disease	Glucocorticoids (IV followed by oral prednisolone), IV cyclophosphamide × 6 then azathioprine	10 Months	Skin lesions on lower extremities	HIV negative, HHV-8 positive	Discontinuation of azathioprine, decrease glucocorticoid dose	Ocular disease flared requiring treatment with interferon alpha, both diseases in remission
Schulhafer et al. [31]	61/M	IgA vasculitis	Glucocorticoids (intravenous prednisolone, oral prednisone), chlorpropamide	6 Months	Skin lesions trunk	HIV status not provided, HHV-8 status not provided	Decrease in prednisone	Regression of KS, IgA vasculitis flared requiring repeat prednisone treatment followed by discontinuation
Vincent et al. [33]	79/F	Leukocytoclastic vasculitis	Glucocorticoids (oral prednisone)	3 Months	Skin lesions on trunk, upper and lower extremities	HIV status not provided, HHV-8 positive	Not provided	Not provided

*F* Female, *GPA* granulomatosis with polyangiitis, *HHV-8* human herpesvirus 8, *HIV* human immunodeficiency virus, *IV* intravenous, *KS* Kaposi sarcoma, *M* male, *MPA* microscopic polyangiitis, *MPO* myeloperoxidase, *PR3* Proteinase 3

Both the cellular and humoral arms of the immune system have been implicated in the control of KS. Immunosuppression is a common theme noted in KS, whether due to innate problems of host immunity, or, due to factors that lead to induced immunosuppression [38]. For instance, the rate of KS in acquired immunodeficiency syndrome (AIDS) patients is inversely proportional to the CD4 count [38]. In non-AIDS associated KS, based on our review of the literature, in patients with rheumatic conditions, glucocorticoids appear to be a consistent risk factor for KS irrespective of other immunosuppressive therapy [39]. Several potential mechanisms have been proposed to explain the association of KS from immunosuppression, including higher expression of chemokine receptors and growth factors, or culprit viral genes. However the data is limited and information is extrapolated from the post-transplantation KS literature [40]. Other possibilities could be the effects of glucocorticoids on lymphocyte depletion [22]. Some studies have found a direct effect of glucocorticoids in stimulating the development and growth of KS [41, 42]. Exogenous glucocorticoids can stimulate the proliferation of spindle cells in KS by upregulation of glucocorticoid receptors [41, 42]. They can also cause direct activation of HHV-8 [41, 42].

Finally, B-cells are latent reservoirs of HHV-8 [6]. While all reported cases of KS in AAV to date have been in patients treated with cyclophosphamide, azathioprine or mycophenolate mofetil, how the increased use of rituximab will affect risk of KS in AAV remains unclear. A recent report included 5 patients who developed KS after treatment with rituximab for their autoimmune conditions (none with AAV) [43]. All were on treatment with glucocorticoids (prednisone dose 10 mg to 35 mg). Time from rituximab to HHV-8 ranged from 3 to 11 months. Four of 5 patients had cutaneous manifestations but gastrointestinal, lung, urogenital disease, pleural effusion and lymphoma were also reported [43]. Two patients required treatment with radiation or chemotherapy [43].

In summary, despite immunosuppression in vasculitis, KS appears to be a rare complication of therapy. It is important to recognize KS as an infectious complication in patients with AAV. The violaceous, nodular lesions in KS, can be mistaken for the palpable purpura from cutaneous vasculitis which also affect the upper and lower extremities [44]. The majority of the cases were within the first year of treatment and the skin was the most frequently affected organ in KS. Glucocorticoid therapy appears to be an important risk factor. Lowering immunosuppression, especially glucocorticoids appears to be beneficial in causing regression of KS. However, this can be challenging since decreasing immunosuppression to help KS could potentially result in recurrence of the underlying systemic vasculitis. A multi-disciplinary approach is important along with individualizing the decision to lower immunosuppression with the possibility of relapse of vasculitis.
